# Narrative exposure therapy for PTSD increases top-down processing of aversive stimuli - evidence from a randomized controlled treatment trial

**DOI:** 10.1186/1471-2202-12-127

**Published:** 2011-12-19

**Authors:** Hannah Adenauer, Claudia Catani, Hannah Gola, Julian Keil, Martina Ruf, Maggie Schauer, Frank Neuner

**Affiliations:** 1 Department of Psychology, University of Konstanz, Konstanz, 78457, Germany; 2 Department of Psychology, Bielefeld University, Bielefeld, 33501, Germany; 3 Department of Psychology, University of Ulm, Ulm, 89081, Germany

## Abstract

**Abstract:**

**Background**

Little is known about the neurobiological foundations of psychotherapy for Posttraumatic Stress Disorder (PTSD). Prior studies have shown that PTSD is associated with altered processing of threatening and aversive stimuli. It remains unclear whether this functional abnormality can be changed by psychotherapy. This is the first randomized controlled treatment trial that examines whether narrative exposure therapy (NET) causes changes in affective stimulus processing in patients with chronic PTSD.

**Methods**

34 refugees with PTSD were randomly assigned to a NET group or to a waitlist control (WLC) group. At pre-test and at four-months follow-up, the diagnostics included the assessment of clinical variables and measurements of neuromagnetic oscillatory brain activity (steady-state visual evoked fields, ssVEF) resulting from exposure to aversive pictures compared to neutral pictures.

**Results**

PTSD as well as depressive symptom severity scores declined in the NET group, whereas symptoms persisted in the WLC group. Only in the NET group, parietal and occipital activity towards threatening pictures increased significantly after therapy.

**Conclusions**

Our results indicate that NET causes an increase of activity associated with cortical top-down regulation of attention towards aversive pictures. The increase of attention allocation to potential threat cues might allow treated patients to re-appraise the actual danger of the current situation and, thereby, reducing PTSD symptoms.

**Registration of the clinical trial:**

Number: NCT00563888

Name: "Change of Neural Network Indicators Through Narrative Treatment of PTSD in Torture Victims" ULR: http://www.clinicaltrials.gov/ct2/show/NCT00563888

## Background

A cardinal characteristic of anxiety disorders is the so-called attentional bias [1,2], which refers to alterations of attentional processes in response to emotional, especially threat-related, stimuli. The attentional bias of subjects with trait anxiety [3,4] as well as anxiety disorders [5,6] including PTSD [7] has recently been described as a 'vigilance-avoidance reaction'. This model describes the tendency to initially attend to threat before this stimulus is avoided in a subsequent reaction. It is hypothized that while aversive cues evoke a rapid response, anxious subjects subsequently initiate attentional avoidance as an attempt to alleviate the fear reaction [3]. Thereby, this model can reconcile the apparently contradicting findings of an increased allocation of attentional resources *towards *aversive stimuli in PTSD that has been found in some studies [8,9], as well as an attentional bias *away *from threat reported elsewhere [10,11]. A recent magnetoencephalographic study indicated that the vigilance-avoidance pattern in PTSD consists of two distinct processes that are temporally and spatially dissociated [12]. PTSD patients showed a hyper-reaction to threatening stimuli in the ventrolateral prefrontal cortex as early as 130 ms after stimulus onset, which was followed by an attentional avoidance reaction in occipital regions starting at 200 ms after stimulus onset. Catani et al. [11] found that the avoidant reaction to aversive pictures in PTSD, indicated by a reduced activity in the left occipital lobe, remained stable for at least the first four seconds after stimulus onset. This reduced activity in brain regions that are associated with elaborate visual processing, was interpreted as disengagement from a detailed visual stimulus exploration [11]. Confronted with potential threats, subjects with anxiety disorders seem to focus on the initiation of a rapid flight reaction rather than concentrating on the attentive evaluation of the threat cue [7]. As it is plausible to assume that such a mechanism can contribute to the maintenance of anxiety symptoms, it could be a promising target for therapeutic interventions. However, the early onset of both the transient vigilant response as well as the prolonged avoidant reaction indicates that both reactions are automatic, stimulus driven bottom-up processes, which cannot be intentionally altered by verbal instruction.

Accumulating evidence from treatment trials demonstrates that PTSD can be effectively treated with psychotherapy. A large number of randomized controlled trials have shown that variants of trauma-focused cognitive behavioral therapy (CBT) are the most successful interventions for the treatment of PTSD [13,14]. The active mechanism of therapeutic change is thought to be the imaginal exposure to the episodic memory of the traumatic event. In this procedure, the patient is instructed to overcome avoidant strategies and to narrate the traumatic experience in detail. The repeated re-experiencing is thought to reconstruct the distorted autobiographic memory of the trauma [15] and to change pathological mechanisms of fear processing.

Little is known about the neurobiological foundations of psychotherapy for PTSD. Three uncontrolled, small-scale studies have examined neuronal changes over the course of psychotherapy. While no changes were found on a structural level [16,17], Felmingham and colleagues (2007) [18] reported a change of activity of basic fear processing mechanisms in eight subjects who underwent CBT for PTSD. In particular, after therapy, amygdala activity declined while the activity of the anterior cingulate cortex, which is associated with a top-down influence on fear processing, increased. As this study lacked a control group however, brain changes could not be causally linked to psychotherapy. To our knowledge, there are only two randomized trials that assessed functional changes of neural correlates in PTSD. These studies reported a decrease of cortical activity in the right anterior hemisphere during exposure to trauma-related pictures [19] and the right middle frontal gyrus during trauma script-driven imagery [20].

Although the primary intention of CBT is to target trauma memories rather than to change attentional processes, the interaction of episodic memory retrieval and attention supports the assumption that trauma focused CBT alters memory as well as attention processes. A cortical structure that might play an important role in the context is the parietal cortex. Recently, Cabeza et al. (2008) formulated a theory that connects attentional and episodic memory processes. According to this theory, goal-directed memory retrieval can be regarded as an effortful process that involves voluntary top-down attention towards memory content. These goal-directed attentional processes are driven by parietal structures including the superior parietal cortex [21]. This finding is important in interpreting the role of the parietal cortex in PTSD considering the general assumption of a deficit in top-down regulation of fear in PTSD [22], which may be modified by exposure therapy [18].

In the present randomized controlled treatment trial, we wanted to investigate the influence of trauma-focused therapy on attentional processing of aversive pictures. We compared the effects of trauma-focused CBT with a waitlist control group in a population of severely traumatized victims of war and torture. In both groups, we assessed neurophysiological indicators of attention to threat cues before and after treatment or the corresponding waiting time. We used Narrative Exposure Therapy (NET) as a variant of trauma-focused CBT. NET is a manualized short-term approach that has been adapted to meet the needs of traumatized survivors of war and torture [23]. NET has been shown to be effective in the reduction of PTSD symptoms in various randomized controlled trials [24-27]. The advantage of NET is that rather than combining various techniques such as cognitive methods and skills training, this treatment focuses exclusively on the reconstruction of episodic trauma memory by a detailed and emotional narration of the biography, with particular emphasis on the traumatic events. Therefore, potential effects can be attributed to a specific intervention rather than to a mixture of various therapeutic methods.

We applied magnetoencephalography (MEG) to measure steady-state visual evoked fields (ssVEF) as an indicator of attention processes. Here, we were interested in the processing pattern of threatening cues, which consisted mainly of pictures with trauma-related (war and attack scenes) or generally aversive (mutilations) stimuli. The ssVEF represents an ongoing cortical oscillatory neuromagnetic response elicited by a repetitive visual stimulus that is presented at a certain frequency (e.g., 10 Hz) having the same fundamental frequency as the driving stimulus [28]. One major advantage of the ssVEF technique is that a high signal-noise-ratio can be achieved even with a limited number of trials [29]. This technique has proven its efficiency in several studies investigating higher order attentional mechanisms during the processing of emotional stimuli in healthy participants [29-31], and psychiatric patients [11,32].

We expected that NET would reduce attentional disengagement towards aversive stimuli [11]. Therefore, we expected a relative increase of activity towards threat-related pictures in brain regions associated with elaborate visual processing. Moreover, we aimed to explore potential changes in activity in the parietal cortex as a marker of enhanced top-down regulation of attention as well as episodic memory.

## Results

### Clinical data

Within the sample of study completers, the Time × Treatment interaction revealed a significant difference of the development of both groups with respect to the CAPS score (F(1, 17) = 34.99, p < .001). In patients treated with NET, PTSD symptom severity significantly declined at the 4-months posttest (p < .001) while the WLC group showed no significant improvement (p = .11). Moreover, an interaction effect for Time × Treatment was found for the Hamilton Depression Rating Scale (F(1, 17) = 13.2, p < .005). The NET group reported significantly less depressive symptom severity at posttest (p < .001) while depressive symptoms stayed unchanged in the WLC group (p = .99). Table 1 provides an overview of PTSD and Depression severity scores at pre- and posttest for each treatment group. Four months after therapy, almost half of the NET group (45.5%) did not fulfill the diagnostic criteria of PTSD anymore. All patients of the WLC condition still met diagnosis of PTSD after matched waiting periods. In the NET group, within-treatment effect sizes were d = 2.21 for the CAPS score and d = 1.56 for the HAM-D score. In the WLC condition, effect sizes were d = -0.97 for the CAPS score and d = -0.07 for the HAM-D score.

**Table 1:** Changes of PTSD and depressive symptoms through NET

		pre-test	post-test	*p*
PTSD symptoms				
CAPS score				
NET (n = 11)	*M (SD)*	88.0 (12.5)	52.8 (18.8)	< .001
WLC (n = 8)	*M (SD)*	72.0 (13.8)	87.9 (18.5)	n.s.
Depressive symptoms				
HDRS score				
NET (n = 11)	*M (SD)*	25.8 (7.9)	14.9 (5.5)	< .001
WLC (n = 8)	*M (SD)*	27.4 (5.6)	27.9 (7.4)	n.s.

Note: Change scores were analyzed with repeated measures ANOVAs.

Changes in medication status from pre- to posttest were little: At posttest, only two treated patients still took antidepressants (compared to four at pretest) and none still took anxiolytics (compared to one at pretest). At posttest, four WLC-patients were medicated with antidepressants (compared to two at pretest) and one was medicated with neuroleptics (compared to none at pretest).

### Minimum norm estimates (MNE)

**Figure 1:**
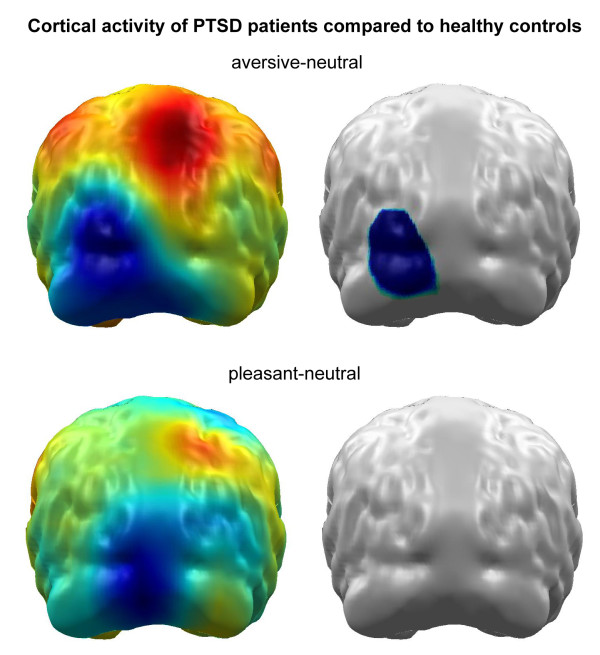
**Difference maps (PTSD minus healthy controls) of cortical activity following aversive (upper panel) and pleasant picture presentation (lower panel)**. The right panel indicates statistically significant brain regions revealed by permutation statistics. Cortical activity following neutral picture presentation was used for normalization. We found significantly reduced activity following aversive picture presentation in the left occipital cortex in PTSD patients compared to healthy controls. No differences of the cortical activation were found between healthy controls and PTSD subjects in following pleasant pictures.

In a former study, we found a significant reduced activity towards aversive pictures in the left occipital cortex in PTSD patients compared to healthy controls [11]. As we found no differences of the cortical activation between healthy controls and PTSD subjects in response of pleasant pictures, only changes in the neural activity for the unpleasant picture are reported here. Healthy controls, as mentioned in Figure 1, overlap to a great extent with the sample reported in the Catani et al. sample.

**Figure 2:**
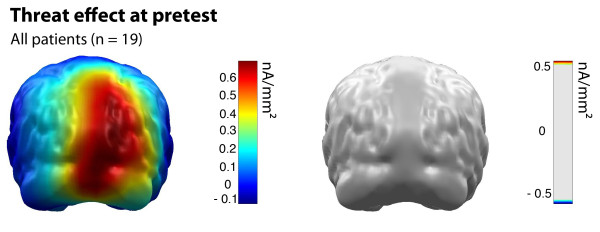
**Threat effect (cortical activation following aversive picture presentation) for all patients at pretest**. Color bar indicates minimum norm source strength (left). Permutation statistics revealed no significant difference between the NET and the WLC group (right). Cortical activity following neutral picture presentation was used for normalization.

At pretest, cortical source activation towards aversive pictures did not differ between NET and WLC group at any dipole location (see Figure 2).

**Figure 3:**
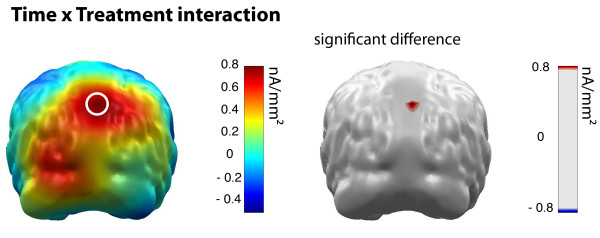
**Threat effect (cortical activation following aversive picture presentation) group differences in pre to post treatment change**. Color bar indicates minimum norm source strength (left). The circle marks significant interaction between the NET and WLC group and pre to post treatment time as indicated by permutation statistics (right). Cortical activity following neutral picture presentation was used for normalization.

**Figure 4:**
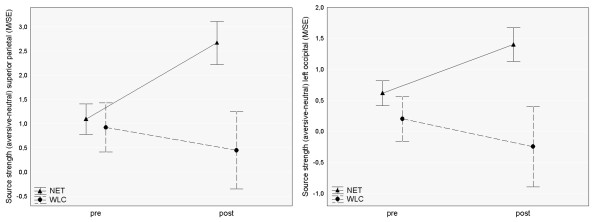
**Pre to post treatment time course of threat effect (cortical activation following aversive picture presentation) in parietal regions (left) and occipital regions (right) for NET and WLC patients**. Ordinates indicate minimum norm source strength. Cortical activity following neutral picture presentation was used for normalization.

With respect to the Time × Treatment interaction, the permutation test revealed a significant effect in the superior parietal cortex (dipoles 47 & 48), which is shown in Figure 3. To illustrate the effects, unpaired t-tests were calculated within this region of interest for both recording sessions (pretest: t = 0.31, p = .76, posttest: t = 2.58, p = .02). Means of the Time effect in occipital regions and the Threat effect in parietal regions are shown separately for NET and WLC patients in Figure 4.

**Figure 5:**
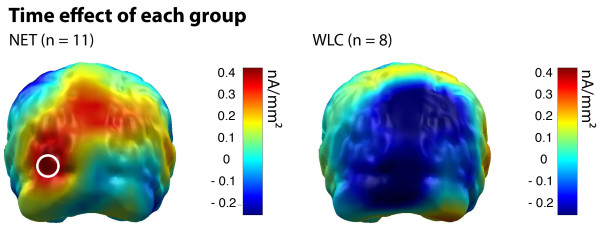
**Pre to post treatment time effect for NET (left) and WLC (right) patients**. Color bar indicates minimum norm source strength following aversive picture presentation. The circle marks significant difference between pre and post treatment time as indicated by permutation statistics. Cortical activity following neutral picture presentation was used for normalization.

Permutation statistics calculated within each group resulted in a significant Time effect, only in the NET group. Patients treated with NET showed an increase of cortical activity towards threatening stimuli at left occipital brain regions (dipoles 91 & 92) from pre- to posttest. This effect was confirmed by paired t-tests (NET: t = -3.35, p = .007). In the WLC group, cortical source activity did not differ in this cortical region between pretest and posttest (t = 0.58, p = .58). Figure 5 shows Time effects for each group separately.

## Discussion

The present randomized controlled treatment trial shows that Narrative Exposure Therapy was effective for the treatment of PTSD symptoms and comorbid depression in traumatized survivors of war and torture. Furthermore, NET changed neural correlates of the processing of aversive stimuli. Specifically, treatment increased the activity of the superior parietal cortex. In addition, patients treated with NET showed an augmentation in occipital activation. These results point at potential neuronal mechanisms underlying effective psychotherapy for PTSD.

After therapy, treated PTSD patients showed a significant increase in parietal activity towards aversive pictures compared to the Waitlist Controls. Even though some studies point to the involvement of the parietal cortex in PTSD [33-35], until recently, no consistent theory exists about its specific role in this disorder. The parietal cortex is traditionally associated with attentional processes [36]. Recent studies, however, showed that the parietal cortex is also relevant in episodic memory retrieval [37,38]. It can be speculated that the increase of parietal activity in treated patients in the present study, represents voluntary top-down episodic memory search that was trained through the procedure of NET, which requires the detailed access to previously avoided episodic memory content. It seems that after the perception of a potentially threatening cue, this voluntary memory search is helpful in evaluating the dangerousness of the stimulus on the basis of previous experiences including the traumatic events. This assumption is in line with current theories of emotional processing in PTSD that attribute changes in exposure therapy to the increasing ability to disentangle past experiences and current threat [23]. Accordingly, successful therapy counteracts attentional avoidance and enhances efficient memory retrieval so that patients are able to identify aversive stimuli as reminders of past experiences rather than as currently dangerous.

Furthermore, a significant increase in activity in left occipital brain regions during aversive stimulation from pre- to posttest was only found in the NET group. Several studies have reported reduced cortical reactivity to threat cues in PTSD patients than non-traumatized control subjects in posterior brain areas. Catani et al. (2009) [11] demonstrated that PTSD patients showed a smaller affective modulation of occipital regions in response to aversive pictures than the control participants. Moreover, Felmingham et al. (2003) [39] also found a reduced ERP signal in PTSD in response to affective faces. These results were interpreted as attentional disengagement or cognitive avoidance of aversive stimuli in PTSD. The finding that only treated patients showed an increase of activity in occipital brain regions after therapy can be interpreted as an enhancement of visual processing of threat cues. As the increase of occipital activity, however, did not reach significance in the Group × Time interaction in the permutation analysis, we have to be cautious about the interpretation of this result. Future studies are needed to replicate our findings with larger sample sizes.

Interestingly, the increase of occipital activation in the NET group was only present in the left hemisphere. This unilateral enhancement of cortical activity in treated patients is consistent with recent studies finding alterations of lateralization in PTSD [19,40]. While the left hemisphere is associated with approach motivation and verbal processing, the right hemisphere is associated with withdrawal motivation and nonverbal, perceptual processing [41]. In PTSD, trauma memory retrieval is mainly accompanied by right hemispheric activation explaining the nonverbal nature of intrusions and the fragmented, incoherent nature of trauma narratives [42,43]. According to Brewin's dual representation theory of PTSD, our finding of increased left hemispheric activation in posterior areas after therapy might reflect the activation of higher order, verbally mediated processes that reduce intrusions [43].

There are a number of limitations of the present study, which illustrate the challenges of studying neural correlates in a sample of severely traumatized participants. One limitation is the loss of MEG data sets of 15 participants after randomization procedure. These dropouts were mostly related to participants' refusal of MEG assessment, poor quality MEG data, and participants' inability to complete the study as a result of deportation due to a denial of asylum by the German authorities. Importantly, these participants did not differ in any clinical variable compared to the study completers. Moreover, none of the participants dropped out of NET treatment voluntarily. Compared to average dropout rates of 20% during ongoing therapy reported in treatment studies using exposure and non-exposure treatments of PTSD [44,45], this finding is particularly noteworthy and shows that NET is well tolerated even by severely traumatized refugees with an unstable asylum status. However, we cannot rule out that with a larger sample size we might have found other involved brain areas. Another limitation of this study is that a significant number of patients took antidepressant and neuroleptic medication. However, as there were no differences between treatment groups with respect to intake of medication, pharmacological effects as an alternative explanation for NET induced changes are improbable. While we found a simultaneous reduction in PTSD, depression as well as neuronal indicators of the attentive system, we are not able to interrelate these changes on an individual basis due to our small sample size. Larger trials are needed to test whether the change of the attentive system is causally related to a reduction of PTSD as assumed by our theoretical foundation. Moreover, without an active control condition, we cannot rule out the contribution of unspecific treatment ingredients for the causation of any effects. Finally, based on the results of the present study, we cannot determine whether the cortical activity reflects a specific response towards trauma-related stimuli or towards high arousing aversive pictures in general. As the majority of pictures showed human attacks, war scenes or injuries, most of them might have triggered memories related to the individual traumatic experiences. It remains a challenging task for future investigations to disentangle these responses.

## Conclusion

In summary, our findings indicate that Narrative Exposure Therapy causes an increase of parietal cortical activity related to the processing of aversive pictures that can be interpreted as a re-establishment of cortical top-down regulation of attention towards these stimuli. NET seems to induce an improvement in episodic memory retrieval that prevents treated patients from being overwhelmed by their own traumatic memories when confronted with aversive or threatening stimuli. Patients treated with NET are able to rely on more elaborate episodic memory contents. They are therefore enabled to re-appraise the direct danger of the current situation, thereby interrupting their former pathological defensive reaction.

## Methods

### Participants

**Figure 6:**
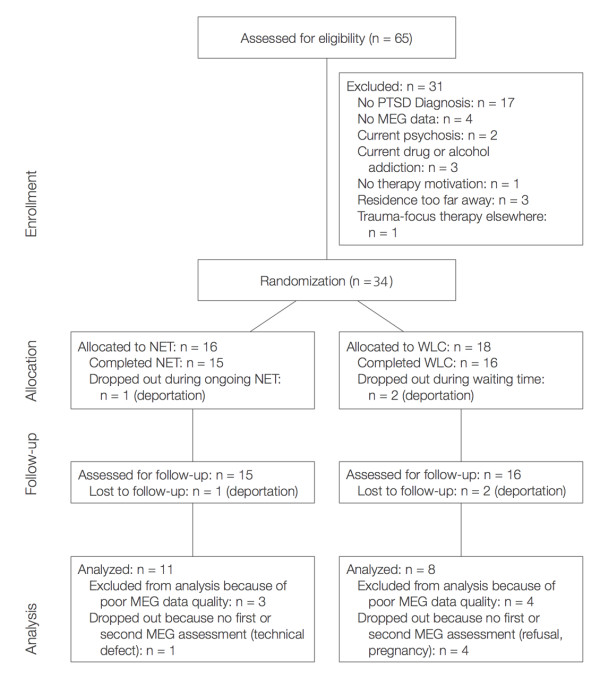
**Flowchart of study participants**. NET indicates Narrative Exposure Therapy, WLC indicates waitlist control condition.

The study was carried out at the Psychological Research and Outpatient Clinic for Refugees at the University of Konstanz. Participants were refugees and asylum seekers who were referred for diagnostic examination or treatment by general practitioners, aid organizations, lawyers, or judges. Inclusion criteria were a history of organized violence or persecution and current PTSD diagnosis according to DSM-IV. Exclusion criteria were current psychosis, substance or alcohol dependence. Thirty-four participants who fulfilled inclusion criteria (see flowchart Figure 6) were randomly assigned to either a treatment (NET) group (n = 16) or a Waitlist Control (WLC) group (n = 18). Age ranged from 16-46 years in the treatment completers group and from 16-56 years in the intention to treat group. A DSM-IV affective disorder (Major Depression or dysthymia) was diagnosed in 68.8% of participants in the NET group and 94.4% of participants in the Waitlist Control group. Participants who completed therapy as well as the MEG examination at posttests and produced MEG data sets (pre and post) that could be included in the final analysis were defined as 'study completers' (SC) (see Table 2).

**Table 2:** Pre-treatment demographic and clinical characteristic of study participants

		Intention to Treat			Study Completer		
		**NET (n = 16)**	**WLC (n = 18)**	***p***	**NET (n = 11)**	**WLC (n = 8)**	***p***

Demographic Data							
Sex				0.97			0.31
female	*N (%)*	7 (43.8)	8 (44.4)		3 (27.3)	4 (50.0)	
male	*N (%)*	9 (56.3)	10 (55.6)		8 (72.7)	4 (50.0)	
Age	*M (SD)*	30.3 (9.2)	36.4 (9.9)	0.09	28.6 (8.6)	33.6 (9.8)	0.25
Education (ys school)	*M (SD)*	8.7 (3.3)	7.8 (3.3)	0.51	8.9 (3.4)	7.9 (2.7)	0.51
Regions of Origin				0.15			0.65
Middle East	*N (%)*	8 (50.0)	11 (61.1)		6 (54.6)	4 (50.0)	
Central East	*N (%)*	2 (12.5)	1 (5.6)		1 (9.1)	1 (12.5)	
The Balkans	*N (%)*	-	3 (16.7)		-	1 (12.5)	
Africa	*N (%)*	6 (37.5)	2 (11.1)		4 (36.4)	2 (25.0)	
Asylum Status (insecure)	*N (%)*	14 (87.5)	18 (100.0)	0.12	9 (81.8)	8 (100.0)	0.20

Clinical Data							
Events (numbers)							
CAPS Event-Types	*M (SD)*	7.4 (2.3)	6.6 (2.0)	0.31	7.0 (2.3)	6.5 (2.1)	0.71
War&Torture-Types	*M (SD)*	11.3 (5.6)	9.4 (4.7)	0.29	11.4 (6.7)	7.6 (4.9)	0.16
Clinical Symptoms							
CAPS Score	*M (SD)*	89.5 (14.7)	80.1 (16.5)	0.16	88.0 (12.5)	72.0 (13.8)	0.04
HDRS	*M (SD)*	27.3 (8.1)	27.3 (6.9)	0.74	25.8 (7.9)	27.4 (5.6)	0.77

Study Completers were called only those participants, who completed therapy and produced MEG data sets (pre and post) of good quality that could be included in the final analysis.

Note: For pair-wise group comparisons of continuous variables, Mann-Whitney *U T*ests were used; differences of categorical variables were evaluated by applying χ^2^-Tests for independence.

### Procedure

The Ethical Committee of the University of Konstanz approved the study protocol and all participants gave written informed consent. The initial assessment consisted of an extensive structured clinical interview and a MEG examination. Clinical psychologists with expertise in the examination of refugees with PTSD carried out the diagnostic interviews with the help of interpreters. MEG recordings took place one week after clinical examination to avoid any possible influence of the diagnostic interview. Participants that fulfilled the inclusion criteria were randomized into the two groups using a computer-generated list of random numbers. Treatment consisted of 12 therapy sessions on a weekly or biweekly basis. On average, treatment sessions lasted 108 (SD = 17.0) minutes. Treatment adherence was monitored by means of regular supervision. Furthermore, all testimonies written down during NET treatment by the therapists (biographical narrations usually exceeding 8000 words) were checked for indicators of vividness and consistency to ensure proper application of NET. No major deviations from treatment protocol were detected.

Posttests with the NET patients were scheduled 4 months after the end of therapy. For the participants in the WLC group, the time spans between pre- and posttests were individually matched with the NET group. Posttests included the same instruments as used in the pretest and were carried out by interviewers who were blind to treatment condition. As part of the posttest, the same MEG examination as in the initial assessment was conducted with each patient.

### Clinical Assessment

The clinical assessment included a detailed structured interview regarding demographic data, traumatic experiences and psychiatric diagnoses. For the examination of traumatic life events, we used the *vivo *Checklist of War, Detention, and Torture Events and the Event Checklist of the Clinician Administered PTSD Scale, CAPS [46]. Standardized clinical instruments were used for the determination of DSM-IV diagnoses: The CAPS for diagnosis and quantification of PTSD, the MINI International Neuropsychiatric Interviews, M.I.N.I. [47] for comorbid DSM-IV axis one disorders, and the Hamilton Depression Scale, HDRS [48] for determining the severity of depressive symptoms.

### Neuromagnetic examination

We applied magnetoencephalography to measure steady-state visual evoked field (ssVEFs) during the presentation of standardized affective pictures varying with respect to emotional content. Full details of the neuromagnetic examination procedure including stimulation, procedure, data preprocessing and analysis have been provided previously [11,29] and are described only briefly here.

### Stimuli

Seventy-five coloured pictures were chosen on the basis of their normative ratings from the International Affective Pictures System (IAPS) [49]. Of these, 25 pictures presented threat-relevant events (e.g., mutilations, assaults, weapons), 25 pleasant events (e.g., sports, erotic couples, children) and 25 neutral events (e.g., neutral faces, household objects). Amongst the unpleasant pictures, the majority of stimuli was threat-related with about one third depicting human assaults or aimed guns, another third showing mutilations or dead bodies. Scenes with soldiers or police officers were presented on three slides, and on another three, angry and sad faces were shown. Pictures were presented with a video projector on a white plastic screen attached to the ceiling of the shielded MEG chamber. In each trial, one picture was presented in a flickering mode of 10 Hz for 4 seconds with an inter-trial interval that varied randomly between 6 to 8 s.

After the initial MEG recording, subjects rated each of the 75 affective pictures for emotional valence and arousal using the Self-Assessment Manikin (SAM) scale. Two participants of the WLC group did not complete SAM ratings. Mean SAM ratings of the aversive pictures were 7.9 for the arousal and 1.4 valence category, of the pleasant pictures 3.9 for the arousal and 6.5 for the valence category and of the neutral pictures 3.3 for the arousal and 4.8 for the valence category. Arousal ratings differed between picture categories (F(2, 28) = 90.6, p < .001, ε = .80) with aversive pictures being rated as more arousing than neutral as well as pleasant (p < .001) pictures. There was no significant difference in the arousal rating between pleasant and neutral pictures.

Pictures differed with respect to patients' valence ratings (F(2, 28) = 233.03, p < .001, ε = .73). Overall, pleasant pictures were rated as most pleasant, followed by neutral and finally by aversive pictures (all comparisons p < .001). There were no statistical differences between the treatment groups with respect to their arousal or valence ratings.

### MEG Recording and Data Processing

**Figure 7:**
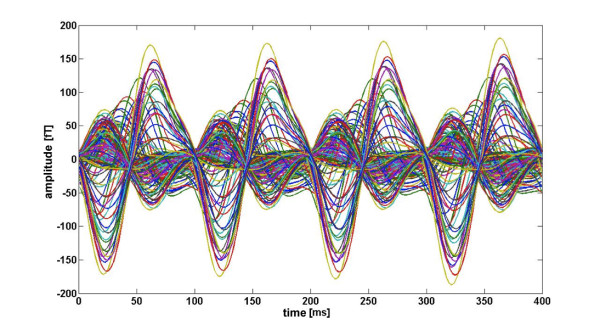
**Four cycles of the 10 Hz steady state response of the 148 MEG channels for one representative participant**.

MEG was recorded continuously and digitized at a rate of 678.17 Hz using a 148-channel whole head magnetometer (MAGNES™ 2500 WH, 4D Neuroimage, San Diego, USA) and an online band-pass filter of 0.1 - 200 Hz. Cardiac and eye artifacts were recorded with a SynAmps amplifier (Neuroscan™) and corrected offline using procedures included in the MEG acquisition software package (Whole Head system software, version 1.2.5; 4D Neuroimaging) as well as algorithms implemented in BESA™ software. In order to determine the position of the head in the MEG-dewar for source localization, individual head shapes and reference points were digitized before the recording session. Offline, MEG data were visually inspected and corrected for movement, cardiac, and eye artifacts as well as for global noise. MEG data were digitally band-pass filtered between 1 Hz and 25 Hz (slopes: 6 and 24 dB/octave, respectively). Finally, trials were baseline-adjusted (500 ms baseline) and averaged over picture category (pleasant, neutral and aversive). For each category average, a moving window averaging procedure was applied [31,50]. To avoid contamination of results with the event related early activity, the initial 400 ms of the picture presentation interval were excluded. A 400 ms window containing four cycles of the 10 Hz flickering stimuli was shifted in steps of 100 ms (one cycle) across the epoch, and the magnetic field data within the shifting windows in the time domain were further averaged. As a result, we obtained for each category, subject, and MEG channel a 400 ms segment of four 10 Hz cycles reflecting an average across 32 sliding windows. Figure 7 shows the four cycles of the 10 Hz steady state response of the 148 MEG channels for one representative participant. These four cycles were submitted to fast Fourier transform (FFT) technique and the extracted real and imaginary parts of the 10 Hz Fourier coefficients were used for source localization.

Using the Matlab-based software EMEGS^© ^[51], the distribution of likely generators of the neuromagnetic activity was estimated by calculating L2 minimum norm solutions based on a spherical one shell (6 cm radius) head model with 197 evenly distributed dipolar sources [52].

### Treatment

Clinical psychologists of the University of Konstanz with expertise in PTSD and NET carried out the treatment according to the manual [53], with the help of a translator if necessary. During the therapy sessions, the patient, assisted by the therapist, constructs a detailed chronological account of his or her own biography. Particular attention is given to traumatic experiences (often events linked to violence and war situations). The autobiography is recorded by the therapist in written form and is corrected and elaborated on each subsequent reading. The therapist writes down the biography and reads it aloud at the beginning of each following session for completion and correction. The aim of the therapy is the reorganization of the generally fragmented report of traumatic experiences into a coherent narrative. During the confrontation with the aversive life events, the therapist asks for current and past emotional, physiological, cognitive, and behavioral reactions, and probes for respective observations. While narrating, the patient is encouraged to relive these emotions. The exposure to the traumatic experience is not terminated during the session until the related fear reaction, presented and reported by the patient, shows a significant diminution. During the last session, the participant receives the written report of the biography [23]. In order to meet the needs of patients with insecure asylum status, the last two sessions were kept flexible to allow patients and therapists to discuss issues related to the current situation.

### Statistical Analysis of Demographic and Clinical Data

Baseline characteristics of the groups were compared to examine the effects of randomization using the Mann-Whitney U test for continuous variables and the Chi-Square test for dichotomous variables. As this study focuses on brain changes through psychotherapy rather than examining the clinical efficacy of the treatment, we restricted all analyses to the sample of study completers. Changes in clinical symptoms were evaluated using repeated-measures ANOVA with Treatment Condition (NET and WLC) as between-factor and Time (pretest and posttest) as within-factor. Greenhouse-Geisser's correction of the degrees of freedom was used where appropriate. The associated epsilon and adjusted p-values are reported. Statistically significant interactions were investigated further by using Tukey's HSD test for post-hoc evaluation of unequal sample sizes. Moreover, clinical significance was estimated by calculating within-treatment effect sizes (Cohen's d) for PTSD (CAPS score) and depressive symptoms (HAM-D score).

### Analysis of MEG Data

For MEG data analysis, brain maps consisting of the minimum norm estimates (MNE) source strengths at 197 dipoles represented the cortical activity induced by the stimulation. The dependent variable in the analysis relates to the cortical activity induced by threatening stimuli. To control for different levels of overall stimulus-driven activity, we subtracted each subject's average activity related to neutral pictures from the average activity in response to threatening pictures ('aversive minus neutral' difference). For simplicity reasons, we refer to the resulting contrast maps as *Threat effects*. As an indicator of the changes induced by the NET group and WLC group, we calculated the differences of the Threat effects between pre- and post test for each group. These contrast maps were defined as *Time effects*. Finally, the Time × Treatment interaction was determined as the contrast map that resulted from the group difference (NET minus WLC) within the Time effects.

The statistical analysis of the MEG data was carried out with permutation statistics rather than t-tests or ANOVAs for several reasons. First of all, due to the small sample size and the skewed distribution of the MEG data the prerequisites of parametric statistics were not fulfilled. In contrast, the permutation test does not require any a priori assumptions about the distribution of data, as it generates a representative subset of a sufficiently large number of permutations of the data to represent the data distribution [54,55]. Secondly, the permutation test avoids the danger of alpha inflation in the comparison of brain maps without the necessity to pre-define regions of interest. The appropriateness of this procedure for analyzing visual-evoked steady-state responses has been demonstrated in previous studies [11,29,32].

Permutation tests were calculated as follows. For each pair-wise comparison, we determined cut-off values for significant differences of the maps at single dipole locations based on 1000 draws. For each draw, the individuals' condition contrast maps were randomly exchanged between groups (NET vs. WLC) and between time points (pre vs. post) to generate data for a random composition with respect to group and time. The maximum as well as the minimum differences at all dipole locations obtained from each draw entered the distributions of 1000 maximum and minimum difference values. The upper and the lower critical values were determined as the 25th lowest and highest values in this distribution (p < .05). Values indicating a significant difference (smaller than the lower and higher than the upper critical values) were plotted onto the MNI brain. For illustrative purposes only, we calculated t-tests for each pair-wise comparison.

## Competing interests

The authors declare that they have no competing interests.

## Authors' contributions

HA carried out the MEG recordings, the data pre processing, source estimation, statistical analysis, she did the clinical interviews and psychotherapies and drafted the manuscript. CC designed the experiment and participated in the statistical analyses, she did the clinical interviews and psychotherapies and helped to draft the manuscript. HG carried out the clinical interviews and psychotherapies and revised the manuscript critically for important intellectual content. JK developed the analytical approach, carried out the data pre processing, source estimation, statistical analysis and revised the manuscript critically for important intellectual content. MR carried out the clinical interviews and psychotherapies and revised the manuscript critically for important intellectual content. MS carried out the clinical interviews and psychotherapies and revised the manuscript critically for important intellectual content. FN conceived the design of the study and statistical analyses, he did interviews and psychotherapies and coordinated and drafted the manuscript. All authors had given final approval of the version to be published.

## Appendix

The numbers of IAPS pictures were as follows. Pleasant: 2190, 2214, 2215, 2383, 2440, 2480, 2516, 2840, 2850, 5130, 5510, 5740, 7035, 7175, 7217, 7491, 7500, 7590, 7595, 7700, 8190, 5830, 5660, 4607, 2209. Neutral: 1722, 2030, 2058, 2165, 2216, 2311, 2340, 2345, 2352, 4599, 4608, 4641, 4653, 4660, 5260, 5700, 8185, 8200, 8380, 8496, 7490, 7130, 5390, 2570, 2410. Unpleasant: 2120, 2900, 3181, 3301, 6190, 6212, 6250, 6312, 6540, 6560, 6831, 6838, 9040, 9181, 9400, 9405, 9415, 9421, 9433, 9911, 6821, 3550, 3530, 2800, 2053.
